# Targeting the Myofibroblastic Cancer-Associated Fibroblast Phenotype Through Inhibition of NOX4

**DOI:** 10.1093/jnci/djx121

**Published:** 2017-08-03

**Authors:** Christopher J Hanley, Massimiliano Mellone, Kirsty Ford, Steve M Thirdborough, Toby Mellows, Steven J Frampton, David M Smith, Elena Harden, Cedric Szyndralewiez, Marc Bullock, Fergus Noble, Karwan A Moutasim, Emma V King, Pandurangan Vijayanand, Alex H Mirnezami, Timothy J Underwood, Christian H Ottensmeier, Gareth J Thomas

**Affiliations:** 1 Cancer Sciences Unit, University of Southampton Faculty of Medicine, Southampton, UK; 2 Genkyotex SA, Plan-les-Ouates, Switzerland; 3 La Jolla Institute for Allergy and Immunology, La Jolla, CA

## Abstract

**Background:**

Cancer-associated fibroblasts (CAFs) are tumor-promoting and correlate with poor survival in many cancers, which has led to their emergence as potential therapeutic targets. However, effective methods to manipulate these cells clinically have yet to be developed.

**Methods:**

CAF accumulation and prognostic significance in head and neck cancer (oral, n = 260; oropharyngeal, n = 271), and colorectal cancer (n = 56) was analyzed using immunohistochemistry. Mechanisms regulating fibroblast-to-myofibroblast transdifferentiation were investigated in vitro using RNA interference/pharmacological inhibitors followed by polymerase chain reaction (PCR), immunoblotting, immunofluorescence, and functional assays. RNA sequencing/bioinformatics and immunohistochemistry were used to analyze NAD(P)H Oxidase-4 (NOX4) expression in different human tumors. NOX4’s role in CAF-mediated tumor progression was assessed in vitro, using CAFs from multiple tissues in Transwell and organotypic culture assays, and in vivo, using xenograft (n = 9–15 per group) and isograft (n = 6 per group) tumor models. All statistical tests were two-sided.

**Results:**

Patients with moderate/high levels of myofibroblastic-CAF had a statistically significant decrease in cancer-specific survival rates in each cancer type analyzed (hazard ratios [HRs] = 1.69–7.25, 95% confidence intervals [CIs] = 1.11 to 31.30, log-rank *P* ≤ .01). Fibroblast-to-myofibroblast transdifferentiation was dependent on a delayed phase of intracellular reactive oxygen species, generated by NOX4, across different anatomical sites and differentiation stimuli. A statistically significant upregulation of NOX4 expression was found in multiple human cancers (*P* < .001), strongly correlating with myofibroblastic-CAFs (*r* = 0.65–0.91, adjusted *P* < .001). Genetic/pharmacological inhibition of NOX4 was found to revert the myofibroblastic-CAF phenotype ex vivo (54.3% decrease in α-smooth muscle actin [α-SMA], 95% CI = 10.6% to 80.9%, *P* = .009), prevent myofibroblastic-CAF accumulation in vivo (53.2%–79.0% decrease in α-SMA across different models, *P* ≤ .02) and slow tumor growth (30.6%–64.0% decrease across different models, *P* ≤ .04).

**Conclusions:**

These data suggest that pharmacological inhibition of NOX4 may have broad applicability for stromal targeting across cancer types.

Over recent years, research has highlighted the contribution of the microenvironment to tumor progression (1), and numerous studies have documented the tumor-promoting role of cancer-associated fibroblasts (CAFs) (2–5). However, these cells remain poorly characterized, and clinically effective treatments targeting CAFs are yet to be developed. This, in part, is due to CAF heterogeneity, which possibly reflects their cell(s) of origin, the tissue in which they develop, and their activation state (6). As a result of this heterogeneity, a single CAF marker is yet to be identified, although several have been proposed in different studies, including α-smooth muscle actin (SMA) (5), fibroblast activation protein-α (FAP) (4), podoplanin (7), and platelet-derived growth factor receptor (PDGFR)-α (8). 

Despite this diversity, CAF are most commonly defined by acquisition of an α-SMA-positive, “activated” myofibroblast phenotype (5). Myofibroblasts share the phenotypic traits of fibroblasts and smooth muscle cells, secreting extracellular matrix (ECM) and generating mechanical tension within tissue through cell contraction (9,10). Myofibroblasts play a key transient role in wound healing but persist in pathological fibrosis and cancer (11), where they contribute to multiple “hallmarks of malignancy” (4,5,12,13). These tumor-promoting properties have led to myofibroblastic CAFs emerging as potential therapeutic targets (14). However, because of a limited understanding of the mechanisms regulating CAF accumulation, effective molecular targeting of this cell population has not yet been achieved.

Previously we have described the tumor-promoting effects of α-SMA-positive myofibroblastic CAF and shown that tumors rich in these cells are associated with poor survival (5,13). The principal aim of this study was to identify a common mechanism regulating differentiation of myofibroblastic CAFs across cancer types in order to develop a strategy to therapeutically target these cells.

## Methods

### Human Tissue Sample Procurement

Archival formalin-fixed paraffin-embedded (FFPE) material was used to construct tissue microarrays (TMAs) from previously described cohorts of head and neck squamous cell carcinoma (HNSCC), esophageal adenocarcinoma (EAC), and early-stage colorectal cancer (CRC) patients (5,12,15,16), composed of triplicate 1 mm cores from randomly selected tumor regions (MiniCore 3, Alphelys - Plaisir, France). Ethical approval was obtained through the UK National Research Ethics Service (NRES; Rec Nos. 10/H0504/32 and 09/H0504/66), and informed consent was obtained from each patient. All tissue collection and storage was handled by a human tissue authority (HTA)–licensed tissue bank.

### Immunohisto/Cytochemistry Staining and Scoring/Quantification

TMAs or whole tissue sections were stained using the automated, commercially available visualization systems Envision FLEX (Dako, Glostrup, Denmark), Dako PT Link (Dako), and Autostainer Link48 (Dako). All antibodies used were optimized to national diagnostic standards (NEQAS). TMAs stained for α-SMA were evaluated using a semiquantitative scoring system described previously (5). Further details can be found in the Supplementary Materials (available online).

### Cell Culture and Reagents

Please see the Supplementary Materials (available online) for details of cell culture conditions and reagents.

### Mouse Models

Mouse experiments were conducted in accordance with the ethical standards outlined in national and international guidelines. All experimental protocols were approved by the authors’ institutional review board (University of Southampton) and by the British Home Office.

Subcutaneous xenograft tumors composed of 5PT cells and human fetal foreskin fibroblasts (HFFF2s) were grown in RAG1^−/−^ mice (n = 14–15/group for shRNA experiments and n = 9–10/group for oral gavage experiments). Subcutaneous isograft tumors comprised of TC1 cells and murine lung fibroblasts (MLFs) were grown in C57/BL6 mice (n = 6/group). Please see the Supplementary Materials (available online) for further details.

### RNA Sequencing and Bioinformatics

Please see the Supplementary Materials (available online) for details of RNA sequencing and bioinformatics analysis.

### Statistical Analysis

Statistical analysis of patient survival rates was carried out with death from cancer as the primary end point; survival time was measured from the date of diagnosis in the HNSCC cohorts and date of surgery in the CRC cohort. Other causes of death were censored at the time of death. Kaplan-Meier plots (with log-rank [Mantel-Cox] tests) and unadjusted Cox proportional hazards models were used to describe the risk of dying from cancer. The proportional hazards assumption was verified by visual assessment of log-minus-log plots and ensuring no statistically significant trend between ranked survival time and Schoenfeld residuals.

Statistical analysis of in vitro and in vivo experiments was carried out on a minimum of three independent experiments or biological replicates. These data are presented as individual points and summarized by mean +/− 95% confidence intervals (CIs) in all figures. The appropriate use of parametric vs nonparametric tests was determined using the D'Agostino and Pearson omnibus normality test, and where sample sizes were too small for this test, a normal distribution was assumed. In cases where statistically significant differences in variance were found between groups or when normalization resulted in 0 variance in one group (eg, in cases where direct statistical comparisons are made with the control sample), Welch’s correction was applied to the calculated *P* value. Statistical tests were carried out using either GraphPad Prism v. 6, R, or IBM SPSS Statistics v. 22. In each case, these are described in the “Methods” section and figure legends. All statistical tests were two-sided, and *P* values of less than .05 were considered statistically significant.

Detailed methods for all other experiments can be found in the Supplementary Materials (available online).

## Results

### Prognostic Significance of Myofibroblastic CAFs

**Figure 1.**
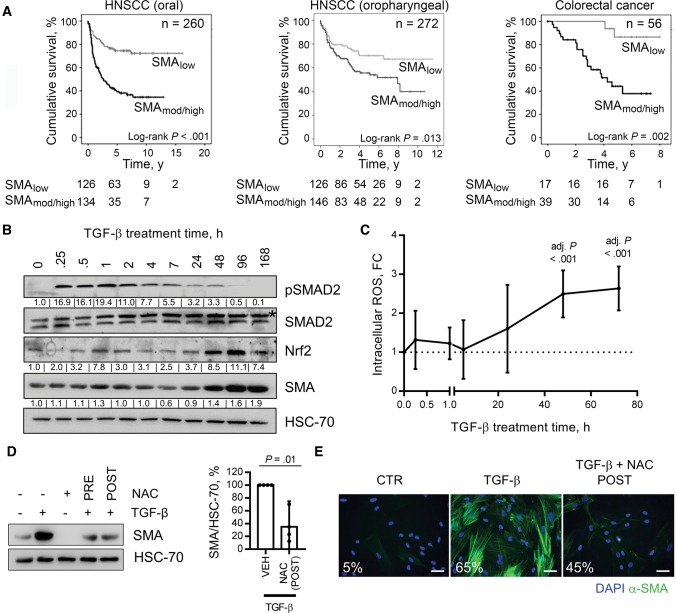
Myofibroblast stroma and survival in cancer and analysis of fibroblast-to-myofibroblast transdifferentiation. **A)** Kaplan-Meier plots of cancer-specific survival rates in patients, with tumor types as indicated (head and neck squamous cell carcinoma), stratified by stromal α-smooth muscle actin (SMA) expression measured by immunohistochemistry. **B and C)** Human fetal foreskin fibroblasts (HFFF2s) treated with transforming growth factor (TGF)–β (2 ng/mL) for the indicated time periods. **B)** Immunoblotting (densitometry quantification shown for α-SMA and Nrf2/HSC-70 and pSMAD2/SMAD2_(Total)_). **C)** Flow cytometry analysis of dichlorofluorescein-diacetate (DCFH-DA) fluorescence. Data are presented as mean +/− 95% confidence intervals from three independent experiments; statistical significance is shown by one-way analysis of variance with Dunnet’s multiple comparison test relative to the untreated control. **D and E)** HFFF2s and primary oral fibroblasts treated with TGF-β (2 ng/mL; 72 hours) +/− NAC (5 mM) administered either one hour prior to TGF-β treatment (PRE) or 24 hours after (POST). **D)** Immunoblotting for α-SMA expression. A representative blot is shown with densitometry quantification (mean +/− 95%CIs from four independent experiments; statistical significance was assessed by two-tailed *t* test with Welch’s correction). **E)** Immunofluorescent cytochemistry staining for α-SMA. The percentage of cells positive for α-SMA stress fibers in each condition is also shown. **Scale bar** represents 50 µm **(E)**. Please see Supplementary Figure 1 (available online) for further information. HNSCC = head and neck squamous cell carcinoma; NAC = N-acetyl cysteine; POST = 24 hours after to TGF-β treatment; PRE = one hour prior to TGF-β treatment; SMA = smooth muscle actin; TGF = transforming growth factor.

To examine the prognostic significance of myofibroblastic CAFs, tumors from cohorts of patients with HNSCC (oral, n = 260; oropharyngeal, n = 271) and early-stage CRC (n = 56) were immunostained for α-SMA and analyzed as described previously (baseline patient characteristics are shown in Table 1) (5,13). In each patient cohort, moderate/high levels of stromal α-SMA identified patients with statistically significantly increased disease-specific mortality (all log-rank *P ≤ *.01; HNSCC [oral]: HR =  3.13, 95% CI =  2.07 to 4.74, *P* < .001; HNSCC [oropharyngeal]: HR = 1.69 , 95% CI = 1.11 to 2.57, *P* = .01; CRC: HR =  7.25, 95% CI =  1.68 to 31.30, *P* = .008) (Figure 1A;Supplementary Figure 1A, available online).

**Table 1.** Baseline clinicopathological features of HNSCC and CRC cohorts*

Clinicopathological feature	HNSCC (oral)	HNSCC (oropharyngeal)	CRC
No. of cases	260	271	56
Mean age at diagnosis (range), y	62 (26–96)	59 (27–92)	73 (39–89)
Mean length of follow-up, y	6.5	4.5	5.25
No. of cancer-related deaths	110	95	22
Gender, No.			
Male	165	197	41
Female	95	74	15
Stage, No.			
I	92	20	4
II	41	31	52
III	12	35	0
IV	115	184	0
Missing	0	1	0
Grade, No.			
Well differentiated	51	3	21
Moderately differentiated	156	100	23
Poorly differentiated	53	166	12
Undifferentiated	0	2	0
Missing	0	1	0
Nodal metastasis, No.			
No	181	54	56
Yes	79	192	0
Missing	0	26	0
Stromal SMA expression, No.			
Low/negative	126	125	17
Mod/high	134	146	39

* CRC = colorectal adenocarcinoma; HNSCC = head and neck squamous cell carcinoma; SMA = smooth muscle actin.

### Investigating the Fibroblast-to-Myofibroblast Transdifferentiation Mechanism In Vitro

Numerous cell types potentially contribute to myofibroblastic CAF accumulation in cancer, but most commonly they are thought to originate from TGF-β-dependent transdifferentiation of local fibroblasts (2–23). In vitro analyses showed that activation of canonical TGF-β signaling in human fibroblasts (SMAD phosphorylation and target gene transcription [*SERPINE1*]) occurred rapidly following TGF-β treatment (15 minutes and one hour, respectively) (Figure 1B;Supplementary Figure 1B, available online), with the full myofibroblast phenotype emerging after 48 to 72 hours (α-SMA upregulation and stress fiber formation) (Figure 1, B and E; Supplementary Figure 1, B and C, available online). Myofibroblast transdifferentiation corresponded temporally with a delayed phase of intracellular reactive oxygen species (ROS) production, maximal between 48 and 72 hours post-TGF-β treatment (shown by increased DCFH-DA fluorescence and upregulation of the antioxidant response protein Nrf2 [*NFE2L2*]) (Figure 1, B and C; Supplementary Figure 1D, available online). Similarly, a delayed phase of intracellular ROS production was observed following other stimuli that promote myofibroblast transdifferentiation (ionizing radiation [IR]) (Supplementary Figure 1G, available online) (24,25).

We found that ROS generation was required for myofibroblast transdifferentiation because the process was suppressed by N-acetyl cysteine (NAC; an anti-oxidant) following TGF-β and IR treatment (Figure 1, D and E; Supplementary Figure 1, E, F, and H, available online). To minimize off-target effects on TGF-β signaling, NAC was also administered 24 hours post-TGF-β treatment, which similarly prevented transdifferentiation (Figure 1, D and E), whereas administering an Alk5 inhibitor (TGF-βRI inhibitor) at this point had no effect (Supplementary Figure 1I, available online). Thus the delayed ROS phase is critical for myofibroblast transdifferentiation, independent of sustained canonical TGF-β signaling.

**Figure 2.**
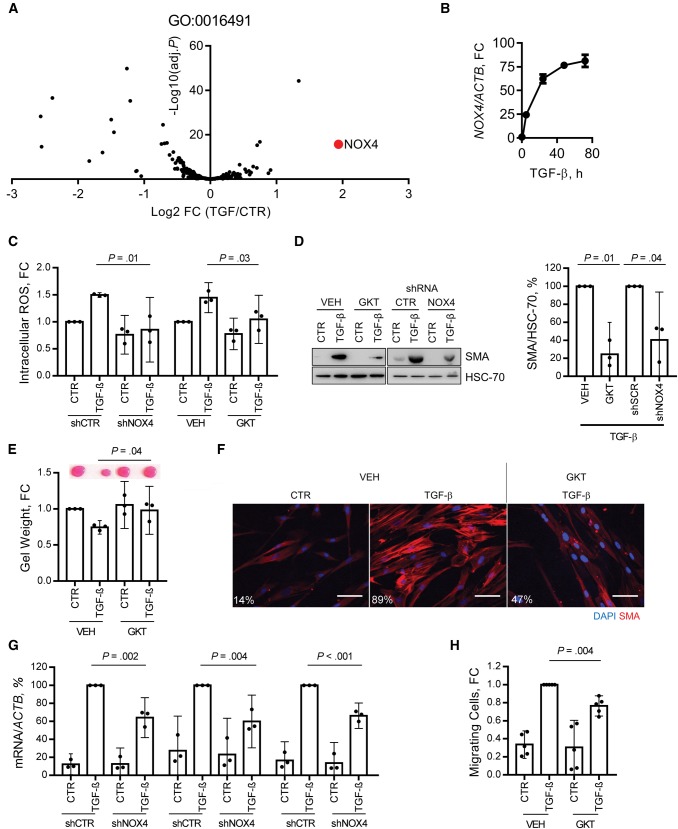
Role of NOX4 in fibroblast-to-myofibroblast transdifferentiation. **A)** Volcano plot showing changes in the expression of genes within the gene ontology term “oxidoreductase activity” (GO: 0016491) in human fetal foreskin fibroblasts (HFFF2s) treated with transforming growth factor (TGF)–β (2 ng/mL, seven days), analyzed by RNA sequencing. NOX4 is identified in **red**. **B)** Quantitative polymerase chain reaction (Q-PCR) analysis of NOX4 mRNA expression following TGF-β (2 ng/mL) treatment in HFFF2s. **C–G)** Analysis of TGF-β-induced ROS and myofibroblast differentiation in HFFF2s following NOX4 inhibition either by GKT137831 (GKT; 20 µM) or lentiviral mediated transduction of shRNA targeting NOX4 (MOI = 20; knockdown confirmed in Supplementary Figure 2F, available online). **C)** Flow cytometry analysis of dichlorofluorescein-diacetate (DCFH-DA) fluorescence at 48 hours from TGF-β treatment (mean +/− 95% CIs from three independent experiments; statistical significance was assessed by two-tailed homoscedastic *t* test). **D)** Immunoblotting for α-smooth muscle actin (SMA) expression. A representative blot is shown with densitometry quantification (mean +/− 95% CIs from three independent experiments; statistical significance was assessed by two-tailed *t* test with Welch’s correction). **E)** Representative images and quantification from collagen gel contraction assays (mean +/− 95% CIs from three independent experiments; statistical significance was assessed by two-tailed homoscedastic *t* test). **F)** Immunofluorescent cytochemistry staining for α-SMA stress fibers. The percentage of cells positive for α-SMA stress fibers in each condition is also shown. **G)** Q-PCR analysis of extracellular matrix–related gene expression (mean +/− 95% CIs from three independent experiments; statistical significance was assessed by two-tailed *t* test with Welch’s correction). **H)** HNSCC cell line (5PT) migration toward conditioned media generated by HFFF2s treated as indicated with TGF-β (2 ng/mL, 72 hours), and/or GKT137831 (20 µM), in a Transwell migration assay (mean +/− 95% CIs from four independent experiments; statistical significance was assessed by two-tailed *t* test with Welch’s correction). **Scale bar** represents 50 µm. (***P < .001). Please see Supplementary Figure 2 (available online) for more information. FC = fold change; TGF = transforming growth factor.

The flavoenzyme inhibitor diphenylene iodonium (DPI) produced a similar effect to NAC (Supplementary Figure 1J, available online), suggesting that the delayed ROS phase was generated enzymatically. We performed RNA sequencing on fibroblasts treated with TGF-β to assess transcriptomic changes during myofibroblast transdifferentiation. Using this data set, the expression of genes within the gene ontology term “oxidoreductase activity” (GO:0016491) was interrogated, showing statistically significant upregulation of NOX4 (false discovery rate, adj. *P* < .001) (Figure 2A). Quantitative (Q-) PCR analysis confirmed persistent upregulation of NOX4 mRNA after approximately five hours of TGF-β treatment (Figure 2B), and immunoblotting showed that increased NOX4 protein expression corresponded temporally with ROS generation (Supplementary Figure 2A, available online). NOX4 upregulation also occurred during myofibroblast transdifferentiation of adult primary fibroblasts isolated from normal oral, skin, and colon tissue biopsies (Supplementary Figure 2, B–C, available online). Pharmacological inhibition of NOX4 (GKT137831) (27) and RNAi-mediated knockdown (shRNA and siRNA) (Supplementary Figure 2, F and G, respectively, available online) abrogated TGF-β-dependent ROS production, myofibroblast transdifferentiation (Figure 2, C–G; Supplementary Figure 2, D–J, available online), and cancer cell migration toward conditioned media from treated fibroblasts (Figure 2H;Supplementary Figure 2K, available online).

NOX4 was also upregulated by alternative myofibroblast transdifferentiation stimuli (IR), and inhibiting NOX4 similarly suppressed ROS generation and myofibroblast transdifferentiation in this context (Supplementary Figure 2, L–N, available online). These data suggested that NOX4 is responsible for the delayed ROS phase, which is critical for fibroblast-to-myofibroblast transdifferentiation.

### Analyzing NOX4 Expression and Myofibroblast Accumulation in Multiple Solid Tumors

**Figure 3.**
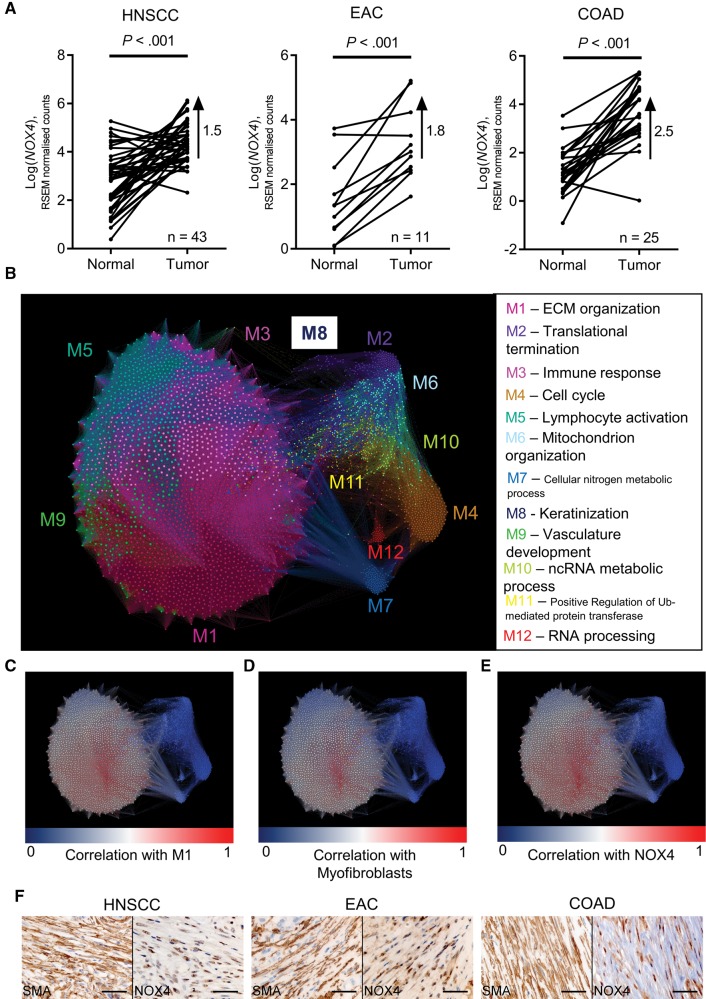
Analysis of NOX4 expression in different cancers. **A–E)** Analysis of RNASeq data from The Cancer Genome Atlas database. **A)** Log-transformed RSEM normalized count (NOX4) expression in patient-matched normal and tumor samples. Statistical significance was assessed using two-sided paired *t* tests. The difference between means is also shown. **B–E)** Network map showing a consensus-weighted gene correlation network, constructed from head and neck squamous cell carcinoma (HNSCC), esophageal adenocarcinoma (EAC), and colon adenocarcinoma (COAD) primary tumor samples. Each node on the network map represents a single gene, and the size of each node represents the connectivity of this gene within the network. **B)** Nodes are colored according to module membership, and the most statistically significantly associated biological function for each module is shown. **C–E)** Nodes are colored according to the degree of correlation (**blue-white-red scale** represents r values increasing from 0 to 1) to the extracellular matrix (ECM) module eigengene **(C)**; to the Mellone_TGF_UR myofibroblast gene signature **(D)** (see Supplementary Table 1, available online, for details); and to NOX4 **(E)**. **F)** Immunohistochemistry staining for NOX4 and α-SMA in serial sections of HNSCC, EAC, and COAD tissue samples (**scale bar** represents 50 µm). Please see Supplementary Figure 3 (available online) for more information. COAD = colon adenocarcinoma; ECM = extracellular matrix; EAC = esophageal adenocarcinoma; HNSCC = head and neck squamous cell carcinoma.

To examine the expression of NOX4 in human tumors, we analyzed RNA sequencing data from multiple cancer types (The Cancer Genome Atlas [TCGA]). A statistically significant upregulation of NOX4 in patient-matched tumor samples compared with normal samples was found in HNSCC, EAC, and colon adenocarcinoma (COAD) (Figure 3A), and similar upregulation was also found in breast carcinoma (BRCA) and lung adenocarcinoma (LUAD; paired two-sided *t* test *P* < .001 for all). To explore NOX4 gene associations, we performed weighted gene correlation network analysis (WGCNA) (28). A consensus network was generated from HNSCC, EAC, and COAD primary tumor samples, identifying 12 modules of highly co-expressed genes conserved between the different tumor types. Four of the modules were associated with stromal biological processes: ECM organization, immune response, lymphocyte activation, and vasculature development (adj. *P* < .001) (Figure 3B and Table 2). A gene signature for TGF-β-activated fibroblasts (myofibroblasts) was derived from the most statistically significantly upregulated genes in TGF-β-treated HFFF2s, identified by RNA sequencing (Mellone_TGF_UR) (Supplementary Table 1, available online) (24); the distribution of these genes within the network was then summarized by the first principal component. Within the consensus network, NOX4 expression positively correlated with those modules relating to stromal processes, and correlated most strongly with the ECM organization module (Table 2). A highly statistically significant positive correlation was found between NOX4 and eigengenes for the ECM module and TGF-β-activated/myofibroblast gene signature in each individual tumor type (Pearson’s *r* > 0.8, adj. *P* < .001 in both cases) (Figure 3, C–E). Furthermore, NOX4 expression was strongly correlated to each of the genes identified as specific markers of CAFs by Tirosh et al. (29) using single-cell RNA sequencing (FAP, THY1, DCN, COL1A1/2, and COL6A1/2/3; NOX4 correlation meta values = 25.7–32.7 for the consensus network and *r* = 0.64–0.86 for individual networks).

**Table 2.** WGCNA module analysis

Module label	Genes in module	Biological process*	Genes in GO term	Genes represented in module	GO term enrichment (FDR-adjusted *P*†)	NOX4 correlation (meta-value)
M1‡	903	ECM organization	409	120	8.95E-59	34.88
M2	631	Translational termination	178	113	3.95E-120	–10.62
M3‡	528	Immune response	1762	190	9.23E-63	21.31
M4	526	Cell cycle	1780	255	2.46E-120	–14.35
M5‡	359	Lymphocyte activation	690	109	7.13E-69	10.96
M6	230	Mitochondrion organization	733	44	1.52E-15	–11.76
M7	177	Cellular nitrogen compound metabolic process	5620	45	.01	–5.11
M8	169	Keratinization	53	15	1.36E-15	–8.22
M9‡	142	Vasculature development	665	48	5.56E-31	23.09
M10	129	ncRNA metabolic process	475	16	2.97E-04	–13.82
M11	126	Positive regulation of ubiquitin-protein transferase activity	98	12	4.89E-09	–9.44
M12	80	RNA processing	826	16	4.98E-04	–7.61

* The most statistically significantly enriched biological process GO term with each module identified in the weighted gene correlation network analysis. ECM = extracellular matrix; FDR = false discovery rate; GO = gene ontology; WGCNA = weighted gene correlation network analysis.

† Two-tailed Fisher exact test with a Benjamini-Hochberg FDR method correction for multiple testing.

‡ Modules attributed to stromal compartments.

**Figure 4.**
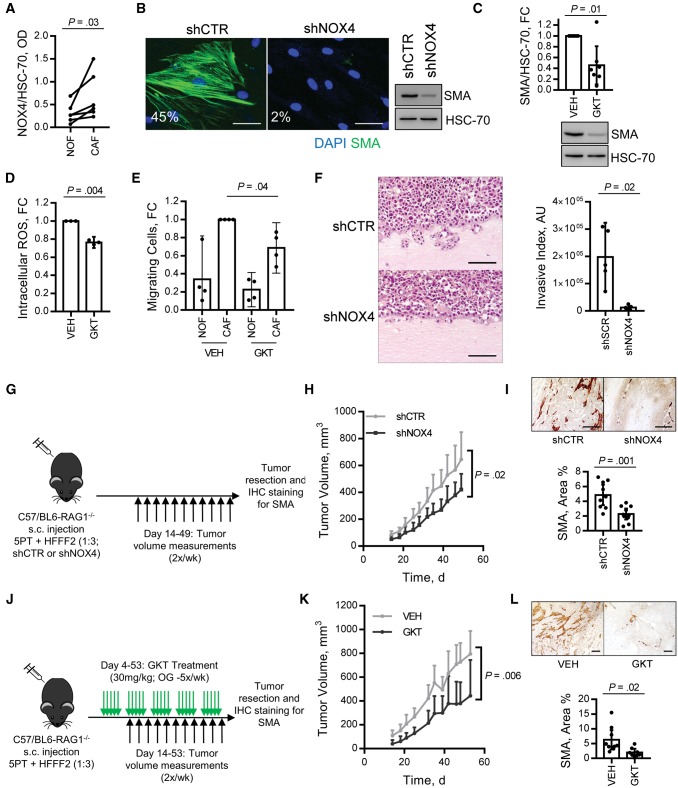
Effect of NOX4 stromal targeting in vitro and in vivo. **A)** Immunoblotting analysis of NOX4 expression in patient matched cancer-associated fibroblasts (CAFs) and normal fibroblasts cultured ex vivo from esophageal tissue biopsies (n = 6; statistical significance was assessed by a two-tailed ratio paired *t* test) (representative blot shown in Supplementary Figure 4A, available online). **B)** Immunofluorescent cytochemistry and immunoblotting for α-smooth muscle actin (α-SMA) expression (the percentage of cells positive for α−SMA stress fibers in each condition is shown; **scale bar** represents 50 µm) in CAFs isolated from head and neck squamous cell carcinoma (HNSCC) transduced with shRNA targeting NOX4 (MOI = 20) (knockdown confirmed in Supplementary Figure 4B, available online). **C–E)** CAFs (n = 7; isolated from HNSCC, n = 1), esophageal adenocarcinoma (EAC; n = 2), colorectal carcinoma (CRC; n = 3) and non–small cell lung carcinoma (NSCLC; n = 1) were treated with GKT137831 (20 µM, 5 days) and analyzed by immunoblotting **(C)** (mean +/− 95% confidence intervals [CIs], n = 7); intracellular ROS assay **(D)** (mean +/− 95% CIs, n = 3 [1-HNSCC and 2-EAC]; and CRC or NSCLC cell lines’ (SW480 and H441) Transwell migration toward conditioned media generated by anatomically matched normal fibroblasts or CAFs (mean +/− 95% CIs, n = 4 [3-CRC and 1-NSCLC]). **C–E)** Statistical significance was assessed by two-tailed *t* test with Welch’s correction. **F–I)** 5PT + human foetal foreskin fibroblasts (HFFF2s) (transduced as per Supplementary Figure 2B, available online) grown in organotypic culture **(F)** or RAG1−/− mice **(G–I)**. **F)** Invasive index was quantified and statistical significance assessed by two-tailed *t* test with Welch’s correction. **H)** Tumor growth curves (mean + 95% CIs, n = 14–15/group; statistical significance was assessed by two-tailed homoscedastic *t* test). **I)** α-SMA immunohistochemistry (IHC) staining representative images and quantification (mean +/−95% CIs, n = 9-10/group; statistical significance was assessed by two-tailed homoscedastic *t* test). **J–L)** 5PT + HFFF2 xenograft tumors were grown in RAG1^−/−^ mice for four days and then treated with GKT137831 (30 mg/kg) by daily oral gavage. **K)** Tumor growth curves (mean + 95% CIs, n = 8/group; statistical significance was assessed by two-tailed homoscedastic *t* test). **L)** α-SMA IHC staining, representative images, and quantification (mean +/− 95% CIs, n = 8/group; statistical significance was assessed by two-tailed *t* test with Welch’s correction). **Scale bars** represent 100 µm unless otherwise stated. Please see Supplementary Figures 4 and 5 (available online) for more information. CAF = cancer-associated fibroblasts; HFFF = human fetal foreskin fibroblasts; IHC = immunohistochemistry; NOF = normal fibroblasts; s.c. = subcutaneous; SMA = smooth muscle actin.

The data indicated that NOX4 is primarily expressed by myofibroblastic CAFs in tumors. Immunohistochemistry staining confirmed that NOX4 and α-SMA were similarly found in stromal regions of HNSCC, EAC, and COAD tissue samples (Figure 3F). Stromal upregulation of NOX4 was also found in data sets of laser microdissected stroma from EAC, LUAD, and BRCA (*P* < .05) (Supplementary Figure 3, A–C, available online) and was statistically significantly upregulated in patient-matched CAFs relative to normal fibroblasts cultured ex vivo from esophageal tissue biopsies (Figure 4A;Supplementary Figure 4A, available online).

To examine a possible causative association between NOX4 expression and myofibroblast transdifferentiation in tumors, we used linear regression analysis. The presence of TGF-β-activated fibroblasts/myofibroblasts or CAFs was measured using principal component analysis (PCA) to summarize the expression of two independent gene expression profiling data sets from TGF-β-treated fibroblasts (from our own data and (30)) and a previously described CAF gene expression signature (Supplementary Table 1, available online) (31). This showed that the level of NOX4 expression accounted for a statistically significant proportion of the variability of these gene signatures in HNSCC, EAC, COAD, rectal adenocarcinoma (READ), invasive breast cancer, LUAD, and pancreatic ductal adenocarcinoma (PDAC) (*r* = 0.65–0.91, *r^2^* = 0.418–0.833, adj. *P* < .001) (Table 3; Supplementary Figure 3, D–F available online).

**Table 3.** Linear regression analysis of NOX4 and myofibroblast/CAF gene signatures

Tumor type	Dependent variable*	n	*r*	*r^2^*	ANOVA	
					*F*	*P*†
HNSCC	Mellone_TGF_UR	498	0.855	0.730	1343.6	2.8E-143
	Verrecchia_TGF_UR	515	0.793	0.629	869.4	1.7E-112
	Mishra_CAF_UR	459	0.846	0.716	1149.9	7.3E-127
EAC	Mellone_TGF_UR	154	0.862	0.744	441.4	8.33E-47
	Verrecchia_TGF_UR	183	0.827	0.684	392.6	3.31E-47
	Mishra_CAF_UR	169	0.859	0.738	471.5	1.66E-50
COAD	Mellone_TGF_UR	234	0.884	0.781	829.4	1.47E-78
	Verrecchia_TGF_UR	282	0.843	0.710	686.2	2.79E-77
	Mishra_CAF_UR	261	0.868	0.754	793.2	8.25E-81
BRCA	Mellone_TGF_UR	969	0.861	0.741	2764.0	8.9E-286
	Verrecchia_TGF_UR	1014	0.794	0.630	1726.4	5.5E-221
	Mishra_CAF_UR	910	0.723	0.522	993.4	6.8E-148
LUAD	Mellone_TGF_UR	447	0.785	0.617	715.4	1.17E-94
	Verrecchia_TGF_UR	481	0.647	0.418	344.2	2.65E-58
	Mishra_CAF_UR	407	0.697	0.486	382.6	1.82E-60
PDAC	Mellone_TGF_UR	169	0.856	0.733	458.3	9.52E-50
	Verrecchia_TGF_UR	173	0.822	0.675	355.7	1.24E-43
	Mishra_CAF_UR	173	0.805	0.649	315.8	1.08E-40
READ	Mellone_TGF_UR	84	0.913	0.833	408.3	1.38E-33
	Verrecchia_TGF_UR	93	0.843	0.710	223.0	3.3E-26
	Mishra_CAF_UR	89	0.847	0.717	220.9	1.34E-25

* Principal component analysis (PCA) was used to summarize the expression of genes associated with myofibroblasts or CAFs, using 3 independent sets of genes previously described to be up-regulated by myofibroblasts/CAFs (detailed in Supplementary Table 1, available online). ANOVA = analysis of variance; BRCA = invasive breast cancer; CAF = cancer-associated fibroblast; COAD = colon adenocarcinoma; EAC = esophageal adenocarcinoma; HNSCC = head and neck squamous cell carcinoma; LUAD = lung adenocarcinoma; PDAC = pancreatic ductal adenocarcinoma; READ = rectal adenocarcinoma.

† A two-sided F test was used to determine overall statistical significance of the linear regression model.

### Testing NOX4’s Potential as a Therapeutic Target

Our results suggested that inhibiting NOX4 may have potential for targeting the myofibroblastic CAF phenotype therapeutically. To test this, CAFs were isolated from human tumors (HNSCC, EAC, CRC, and non–small cell lung cancer [NSCLC]) and treated with GKT137831 or shRNA to inhibit NOX4. This suppressed intracellular ROS production and α-SMA expression, suggesting reversion to a more fibroblast-like phenotype (54.3%, 95% CI = 10.6% to 80.9%, decrease in α-SMA, *P* < .01) (Figure 4, B–D; Supplementary Figure 4, B and C, available online). Consistent with this, NOX4 inhibition also abrogated CAF-dependent tumor cell migration/invasion in Transwell and organotypic culture assays (Figure 4, E and F; Supplementary Figure 4D, available online).

HNSCC (5PT) cells promote fibroblast-to-myofibroblast transdifferentiation in HFFF2s in vitro (Supplementary Figure 4, E–G, available online), which is suppressed by NOX4 inhibition (Supplementary Figure 4G, available online). The 5PT/HFFF2 interaction in vivo, within a xenograft tumor model grown in partially immune-compromised (RAG1^−/−^) mice, also generates an α-SMA-positive myofibroblastic stroma (Figure 4I and 4L) and promotes tumor growth (Supplementary Figure 4H, available online). Using this model, we tested the efficacy of inhibiting NOX4 for preventing myofibroblastic CAF accumulation and tumor progression. Co-injection of HFFF2s transduced with shRNA targeting NOX4 and 5PT cells caused a statistically significant reduction in myofibroblast accumulation (53.2%, 95% CI = 24.1% to 82.2%, *P* = .01) and tumor growth (37.6%, 95% CI = 6.0% to 69.2%, *P* = .02) (Figure 4, G–I). A similar effect was observed by targeting NOX4 pharmacologically; RAG1^−/−^ mice were inoculated with 5PT+HFFF2 xenograft tumors, which were allowed to establish for four days. These mice were then treated with GKT137831 (30 mg/kg by oral gavage), which statistically significantly reduced myofibroblast accumulation (68.4%, 95% CI = 14.6% to 122.3%, *P* = .02) and tumor growth (46.8%, 95% CI = 15.9% to 77.8%, *P* = .006) (Figure 4, J–L; Supplementary Figure 4, H and I, available online). GKT137831 treatment had no statistically significant effect on tumor growth in the absence of co-injected fibroblasts, suggesting that this compound predominantly acts through stromal targeting in this model (Supplementary Figure 4H, available online).

In vivo experiments were also performed using an isograft model (lung tumor model [TC1] + murine lung fibroblasts [MLFs] grown in C57/BL6 mice). To generate a myofibroblastic stroma, MLFs were treated with TGF-β in vitro prior to injection (Supplementary Figure 5A, available online). Similar to the xenograft model, NOX4 inhibition (shRNA) (Supplementary Figure 5, B–E, available online) and GKT137831 (Supplementary Figure 5, F–H, available online) statistically significantly reduced myofibroblast accumulation (79.0%, 95% CI = 66.0% to 92.0%, and 76.3%, 95% CI = 54.1% to 98.6%, respectively, *P* < .001) and tumor growth (64.0%, 95% CI = 30.9% to 97.1%, and 30.6%, 95% CI = 1.9% to 59.3%, respectively, *P* ≤ .04).

## Discussion

The heterogeneous CAF population remains poorly defined; however, our data show that across several human tumor types, the presence of an α-SMA-positive stroma identifies patients with poor survival (5,13,18). Targeting this CAF phenotype is therefore an attractive therapeutic option, and in this study, we show that NOX4 is a common regulator of myofibroblast accumulation in many human cancers that can be targeted pharmacologically to suppress/revert myofibroblastic CAF differentiation and slow tumor growth.

Tumor cells have been shown to induce TGF-β 1-dependent upregulation of NOX4 in breast stromal cells in vitro (32); however, to date, investigation of the role of NOX4 in cancer has predominantly focused on the tumor cell, where a variety of functional effects have been described (33–37). In this study, we show in multiple tumors that NOX4 expression correlates strongly with myofibroblastic CAF accumulation. We found that NOX4 regulates fibroblast-to-myofibroblast transdifferentiation by generating a delayed phase of intracellular ROS, independent of sustained canonical TGF-β signaling. This mechanism is common to fibroblasts derived from different anatomical sites and across different transdifferentiation-promoting stimuli. Furthermore, we show that NOX4 inhibition in CAFs can abrogate the tumor-promoting function of these cells in vitro and in vivo and that this effect can be achieved pharmacologically (using GKT137831), identifying this enzyme as a druggable therapeutic target. Consistent with our findings, NOX4 has been shown to play a role in TGF-β-dependent fibroblast-to-myofibroblast transdifferentiation in several normal tissues (27,38–40), and its inhibition has been shown to attenuate pulmonary and liver fibrosis in preclinical mouse models (27,38,41).

CAFs are becoming increasingly recognized as potential therapeutic targets because of their multifaceted role in tumor progression, and it is hypothesized that their genetic stability makes them vulnerable to targeted therapies (14,42). It remains to be determined, however, how best to do this and in what context because CAF targeting, even if successful, is unlikely to be effective as single-agent therapy. While TGF-β signaling is known to promote myofibroblast differentiation, targeting this pathway in cancer is problematic because it also regulates tumor-suppressive functions, and ubiquitous inhibition has been shown to result in the development of cutaneous keratoacanthomas/squamous cell carcinomas (43,44). Recent clinical trials have highlighted several drugs that improve response to chemotherapy while also (inadvertently) appearing to target the tumor stroma. For example, albumin-bound (nab)-paclitaxel (designed for effective drug delivery of highly lipophilic agents) has been shown to lead to CAF reduction and stromal disruption in PDAC, increasing the efficacy of gemcitabine and improving patient survival rates (45,46). Similarly, the broad tyrosine kinase inhibitor nintedanib (anti-VEGFR, -FGFR, -PDGFR), developed as an angiogenesis inhibitor, increases responses to docetaxel in non–small cell lung cancer patients (47) and has also shown some efficacy in the treatment of idiopathic pulmonary fibrosis (48).

Treatments designed to specifically target CAFs, however, have not been successful clinically. Inhibition of hedgehog signalling and depletion of FAP-positive CAFs have been shown to enhance chemotherapy delivery (49) and antitumor immunity (4) in murine models. However, both strategies have produced disappointing results in phase II clinical trials for treatment of metastatic pancreatic and colorectal cancers, respectively (50–52). These poor clinical results are potentially explained by recent studies that showed that stromal depletion in murine PDAC models can lead to reduced survival (53–55) and that depletion of FAP-positive cells results in cachexia and anemia due to loss of bone marrow–derived stem cells (56,57). Oxidative stress has also been described to play a key role in myofibroblast differentiation, both in tumors and fibrosis (58,59). However, the use of various anti-oxidants to target these effects clinically has proven similarly unsuccessful (60–62) and has even been shown to increase the risk of metastatic spread in a murine melanoma model (63).

Our data suggest that NOX4 inhibition represents a specific method to abrogate the generation of intracellular ROS in fibroblasts, a critical step in myofibroblast differentiation/CAF accumulation. Notably, we found that NOX4 inhibition also can revert α-SMA-positive CAF to a more fibroblast-like cell, suggesting that the CAF phenotype is potentially reversible and not fixed in a terminally differentiated state. The xenograft and isograft subcutaneous tumor models used in this study develop a myofibroblastic and collagenous stroma with similar structural properties to human tumors (18,24); we found that this can be suppressed pharmacologically using GKT137831, a small organic molecule of the pyrazolopyridine dione chemical class, which is a selective inhibitor of NOX1/4 and the first drug in this class of NOX inhibitors to enter the clinic. A recent phase II clinical trial examining its potential for inhibiting renal fibrosis in patients with diabetic nephropathy demonstrated pharmacological effectiveness and an excellent safety profile (NCT02010242). Potential use in cancer has not been considered, but may be considerable.

There are some limitations that must be considered when interpreting these findings; the analysis of myofibroblastic CAF association with survival was carried out using retrospective cohorts, excluding patients lost to follow-up or with insufficient archival material for analysis. These criteria may have introduced some level of bias within the cohort if these groups were characteristically different than those included. In addition, we have only examined the potential for NOX4 inhibition in the context of single-agent therapy, showing that this reduces myofibroblast accumulation and tumor growth. To translate these findings clinically, additional work is required to determine the best approach for combining NOX4 inhibition with other types of therapy, examining the effect of these treatments on tumor progression in more detail (for example, effect on metastatic spread), and using spontaneous/orthotopic murine model systems.

In summary, our data suggest that NOX4 inhibition may have broad applicability for stromal targeting across cancer types. Given the previously described roles for CAFs in promoting resistance to chemotherapy (64,65) and adaptive antitumor immunity (4,66), stromal depletion through NOX4 inhibition may be a useful adjunct to improve response to current chemo- and immunotherapeutics in patients with α-SMA-positive CAF-rich tumors.

## Funding

This work was supported by Cancer Research UK (grant Nos. C21825/A13315 and C115121/A20256) and Wessex Medical Research.

## Notes

The funders had no role in design of the study; the collection, analysis, or interpretation of the data; the writing of the manuscript; or the decision to submit the manuscript for publication.

We thank the University of Southampton Biological Research Facility (particularly Lisa Dunning Tod and Vikki Field) for support with the animal work and the Faculty of Medicine Tissue Bank for providing biopsies for fibroblast isolation.

Studies were designed and planned by GJT, CJH, and MM; experimentation was carried out by CJH, MM, KF, TM, SJF, EH, and DMS; human tissue sample procurement and accompanying patient database construction was carried out by MB, AHM, FN, EVK, and TJU; immunohistochemistry scoring was carried out by KAM, TJU, and GJT; CS optimized the use of GKT137831 in vivo; MM, PV, CJH, SMT, and CHO contributed to RNA sequencing data collection and analysis; CJH, MM, SMT, and GJT analyzed the data; and CJH, MM, SMT, CHO, and GJT wrote the manuscript.

The authors declare no competing financial interests.

RNA sequencing analysis of HFFF2s has been made available using the ArrayExpress archive: https://www.ebi.ac.uk/arrayexpress/ (username: Reviewer_E-MTAB-3101; password: qqfvtsnb).

## Supplementary Material

Supplementary figures and tables
