# Supplementary material for: Targeting the Myofibroblastic Cancer-Associated Fibroblast Phenotype Through Inhibition of NOX4
Source: J Natl Cancer Inst. 2017 Aug 3;110(1):109–20. doi: 10.1093/jnci/djx121 (PMC5903651; doi:10.1093/jnci/djx121)
Supplement: Supplementary figures and tables [file djx121_supp.pdf]

# SUPPLEMENTARY MATERIALS

## Targeting the myofibroblastic cancer-associated fibroblast phenotype through inhibition of NOX4

Christopher J. Hanley[1†], Massimiliano Mellone[1†], Kirsty Ford[1], Steve M. Thirdborough[1], Toby Mellows[1], Steven J. Frampton[1], David M. Smith[1], Elena Harden[1], Cedric Szyndralewiez[2], Marc Bullock[1], Fergus Noble[1], Karwan A. Moutasim[1], Emma V. King[1], Pandurangan Vijayanand[3], Alex H. Mirnezami[1], Tim J. Underwood[1], Christian H Ottensmeier[1], Gareth J. Thomas[1]*.

[1]Cancer Sciences Unit, University of Southampton Faculty of Medicine, Southampton SO16 6YD, United Kingdom.

[2]Genkyotex SA, Chemin des Aulx 16, 1228 Plan-les-Ouates, Switzerland.

[3]La Jolla Institute for Allergy & Immunology, La Jolla, California 92037, USA

*Corresponding Author: Professor Gareth J. Thomas, University of Southampton, Faculty of Medicine, Cancer Sciences Unit, Somers Building, MP 824,Tremona Road, Southampton, SO16 6YD, Tel: 0044-(0)7845 361124, email: G.Thomas@soton.ac.uk

†These authors contributed to this work equally.

## Supplementary Materials and Methods

### Immunohisto/cytochemistry staining and analysis

*Human Formalin Fixed Paraffin Embedded (FFPE) tissue samples*

4µm FFPE sections were mounted on Superfrost+ (Thermo Fisher Scientific, Waltham, Massachusetts) Slides and baked for 30 minutes at 65°C prior to use. Deparaffinisation, rehydration and antigen retrieval was completed for 20 minutes at 97°C using a predefined program on the Dako PT links. Antigen retrieval was performed using the Envision FLEX High pH. Endogenous peroxidase activity was blocked using Envision FLEX blocking reagent (Dako). Primary antibodies were incubated for 20 minutes. Secondary amplification and enzymatic conjugation was completed using Envision FLEX HRP (Dako, 20 mins) and Rabbit Link (Dako, 15 mins for NOX4) reagents as appropriate. Chromogenic visualization was completed with 2x5 minute washes in DAB and counterstaining with Haematoxylin. The following antibodies were used: anti-α-SMA (M0851, Dako); anti-NOX4 (NB110-58849, Novus Biologicals, Abingdon, UK).

*Mouse FFPE xenograft tumours*

Mouse xenograft tumours were stained using the commercially available Mouse on Mouse IHC detection kit (PK-2200, VectorLabs, Peterborough, UK) according to manufacturer's instructions.

*Scoring and Quantification*

TMAs stained for α-SMA were evaluated using a semi-quantitative scoring system described previously according to the extent of stromal positivity (low/negative [<5% stroma positive], moderate [patchy/focal expression, 5–50% stroma positive] or high [diffuse expression throughout the tumour, >50% stroma positive]. Scoring was carried out independently by (GJT, KAM and TJU) blinded to clinical outcome.

Quantification of α-SMA staining in mouse xenograft tumours was carried out using colour thresholding in Fiji (1). 3-5 independent fields of view (FOV) were analysed for each tumour in order to generate a mean value per tumour, which was compared between treatment groups.

*Immunocytochemistry*

Immunocytochemistry staining and imaging were performed as follows. Samples were fixed (4% PFA), permeabilized (0.5% Triton X-100), blocked in BSA (2% *w/v*), incubated with primary antibodies and secondary antibodies conjugated to fluorescent proteins. A DAPI counterstain was used to visualize nuclei.

The antibodies used were as follows: α-SMA (1:750, A2547- SIGMA, Gillingham, UK,); Mouse-IgG Alexa 488 conjugated (1:250, 11017 - Invitrogen, Carlsbad, California); Mouse IgG Alexa 564 conjugated (1:250, 11003 - Invitrogen). The percentage of α-SMA-positive cells was determined in an automated manner using FIJI image analysis software[1]. Briefly, 6-10 randomly selected FOVs were imaged. Within each image individual cells were divided into regions of interest (ROI) using DAPI staining to generate segmented particles. The corresponding α-SMA stained image was then processed by applying a 10 pixel rolling ball background subtraction and despeckling. Thresholding was then carried out to produce a binary image representing stress fibres, using the mean fluorescence intensity value from the positive control sample (TGF-β or untreated CAF) as a cut-off. The positive area fraction in each cell was then measured. Positive cells were defined by an area greater than 10% with α-SMA stress fibre positivity.

## Cell Culture and Reagents

*Cell Culture*

Adult human primary fibroblasts (characterized as cytokeratin-negative, CD31-negative, vimentin-positive) were isolated from normal and cancerous tissue from human colonic,

oesophageal, lung or oral tissue, with ethical approval and informed consent. Murine lung fibroblasts (MLFs) were similarly isolated from C57/BL6 lung samples. Biopsies were suspended in PBS supplemented with 100 U/ml penicillin, 100μg/ml streptomycin, and 0.25μg/ml amphotericin B. The biopsy was divided into pieces (~5mm$^2$), and cultured in DMEM supplemented with 20% foetal calf serum, 100u/ml penicillin, 100μg/ml streptomycin and 292μg/ml L-Glutamine. After 3-4 weeks fibroblasts were detached and cultured as described below.

SW480 and H441 (CRC and NSCLC cell lines; procured from ATCC); human foetal foreskin fibroblasts (HFFF2s) and HEK-293T cells were cultured in Dulbecco's modified Eagle's medium (DMEM) supplemented with 10% FBS (Calbiochem, San Diego, California) and 292μg/ml L-Glutamine. Adult human primary fibroblasts were cultured in DMEM supplemented with 10% FBS (Calbiochem), 292μg/ml L-Glutamine and 1% Penicillin/streptomycin. MLFs were cultured in the same media under hypoxia (3% $O_2$). HNSCC cell line 5PT (2) was cultured in keratinocyte growth medium (KGM), as previously described (3). TC1 (murine lung cancer cells; procured from ATCC) were culture in RPMI supplemented with 10% FBS (Calbiochem) and 292μg/ml L-Glutamine. None of the cell lines used are within those commonly misidentified (International Cell Line Authentication Committee (ICLAC); http://iclac.org/databases/cross-contaminations/). All cell cultures were routinely tested for mycoplasma contamination.

*Pharmacological Inhibitors*

Human Recombinant TGF-β (R&D Systems, Minneapolis, Minnesota) was reconstituted in 4mM HCl, 0.1% (*w/v*) BSA and administered to cells at a dose of 2ng/ml. N-Acetyl Cysteine (NAC; SIGMA) was reconstituted in $H_2O$ and administered at 5mM. Diphenylene Iodonium (DPI; SIGMA) was reconstituted in DMSO and administered at 4μM. The ALK5 inhibitor (inhibits TGF-βRI; Calbiochem) was reconstituted in DMSO and administered at 1μM. GKT137831 (GKT; kindly provided by Genkyotex, Geneva, Switzerland) was reconstituted in

DMSO and administered at 20µM and replenished every 48 hours. $H_2O_2$ (SIGMA) was prepared in $H_2O$ and administered at 1mM for 1 hour, the media was then removed and replaced. For IR treatment cells were exposed to 10Gy $\gamma$-irradiation in suspension.

*RNA interference (RNAi)*

RNAi mediated knockdown was carried out either transiently or stably using siRNA or shRNA respectively. Transient siRNA-mediated knockdown was carried out using Oligofectamine reagent (Thermo Fisher Scientific) as per the manufacturer's instructions, cells were transfected with NOX4 On-Target pool siRNA or non-targeting pool (CTR) siRNA (25nM; Thermo Fisher Scientific).

Stable knockdown was carried out using lentiviral mediated transduction of shRNA. Lentiviral particles were generated as described previously (4). Briefly, lentiviral particles were produced in HEK-293T cells by transient transfection with the pLKO.1 lentiviral vector, pCMVDR8.91 and pMD.G plasmids, using Fu-GENE HD transfection reagent (Promega, Madison, Wisconsin). The resulting viral titre was estimated using a cell viability assay in response to puromycin for each individual fibroblast type (CAF E, HFFF2s and MLFs), to ensure delivery of a comparable viral load between shRNA targeting NOX4 and non-targeting shRNA (CTR). Fibroblasts were then transduced using a multiplicity of infection (MOI) of 20 and cultured in puromycin (0.75mg/ml). Selection was confirmed using a mock transduced flask of cells in parallel.

**Animal Models**

*Xenograft model*

$1x10^6$ 5PT cells ± $3x10^6$ HFFF2s were re-suspended in 150ml of serum free DMEM; 100ml of this mix was injected subcutaneously into the flank of partially immunocompromised B6.129S7-Rag1<tm1Mom>/J ($RAG1^{-/-}$) mice (3-5 months old). Female mice were used for shRNA experiments and male mice for oral gavage experiments. For shRNA experiments

14-15 tumours were analysed per group with shCTR or shNOX4 fibroblasts injected in opposing flanks. For oral gavage experiments 9-10 tumours were analysed per group, mice were randomly assigned to treatment groups prior to tumour inoculation, tumours were injected and allowed to establish for 4 days prior to commencing treatment. GKT137831 (Genkyotex), was prepared daily (5/7 days/week) in 1.2% Methyl cellulose plus 0.8% Polysorbate80 (Sigma), and administered by oral gavage at a 30mg/kg dose.

*Isograft model*

The isograft model was performed similarly to the xenograft model (described above), with $1x10^5$ TC1 cells ± $3x10^5$ murine lung fibroblasts (MLFs) injected into C57/BL6 mice (6-8 weeks old); female mice were used for oral gavage experiments and male mice for shRNA experiments.

*Tumour Volume measurements and processing*

Tumour size was measured using an electronic calliper and calculated using the formula $4\pi/3 \times r^3$ (radius (r) calculated from the average diameter, measured as the tumour width and length) (12). Differences between tumour growth rates were tested for statistical significance using a 2-tailed homoscedastic t-test comparing the area under the curve (AUC) for each individual tumour within a treatment group. Sample sizes were estimated based on the variability observed in preliminary experiments. Animals were excluded from the analysis if the tumour was found to develop intramuscularly rather than sub-cutaneously upon dissection.

Following euthanasia tumours were excised, fixed in 10% formalin and processed to paraffin for IHC.  Fresh tumour was also snap-frozen in liquid nitrogen and used for Western blotting analysis.

**ROS assay**

Intracellular ROS were measured using flow cytometry analysis of dichlorofluorescin di-acetate (DCFH-DA) fluorescence. Cells were incubated for 15 minutes at 37˚C with 5μM DCFH-DA to load cells with the dye; if inhibitors were used, cells were pre-treated for 30 minutes and then added to the DCFH-DA incubation step. Cells were then trypsinized, centrifuged and re-suspended in cold PBS and kept on ice, protected from light; fluorescence was measured on the FL-1 channel of a FACS CANTO II and the mean fluorescence intensity (MFI) was calculated using BD FACS DIVA software.

**Q-PCR**

Real time quantitative PCR analysis was carried out as described previously (5), using SYBR Green reagents (Thermo Fisher Scientific). The primers used were as follows: *ACTB* (forward TGGCACCCAGCACAATGAA, reverse CTAAGTCATAGTCCGCCTAGAAGCA) as a housekeeping gene; *COL1A1* (forward ACGAAGACATCCCACCAATCACCT, reverse AGARCACGTCATCGCACAACACCT); *COL3A1* (forward AATCAGGTAGACCCGGACGA, reverse TTCGTCCATCGAAGCCTCTG); *ACTA2* (forward GACAATGGCTCTGGGCTCTGTAA, reverse ATGCCATGTTCTATCGGGTACTT); *FN* (forward TGTGGTTGCCTTGCACGA, reverse GCTTGTGGGTGTGACCTGAGT). *Hprt1* (forward GTTGGGCTTACCTCACTGCT, reverse TCATCGCTAATCACGACGCT); *Nox4* (forward TGCCCCAGTGTATCAGCATT, reverse CCGGAATCGTTCTGTCCAGT).

**Western Blotting**

Western blotting was carried out as described previously (6), with minimal alterations. RIPA buffer was used for cell lysis from *in vitro* experiments and Laemmli buffer was used for lysis of xenograft tumour samples. Densitometry quantification was performed using FIJI image analysis software (1). The antibodies used were as follows: α-SMA (1:1000, A2547 - SIGMA); NOX4 (1:500, NB110-58849 - Novus Biologicals,); NRF2 (1:500, SC-30915 - Santa Cruz, Dallas, Texas); Palladin (1:500, NBP1-80952 - Novus Biologicals); SMAD2 (1:500,

#3122 - Cell Signaling, Danvers, Massachusetts); pSMAD2 (Ser 465/467; 1:500, #3108 - Cell Signaling); HSC-70 (1:1000, SC-7298 - Santa Cruz,); Mouse-IgG (HRP conjugated; 1:10000, P0260 - Dako,); Rabbit-IgG (HRP conjugated; 1:5000, P0217 - Dako); and Goat-IgG (HRP conjugated; 1:5000, P0449 - Dako).

Invasion assays:

**Transwell Invasion/Migration**

Fibroblast conditioned medium (CM) was prepared in serum fee α-MEM from 72 hours in culture. The collected CM was centrifuged at 2000 rpm, filtered (0.2mm) and normalized according to cell number. This CM was then used as a chemoattractant in the lower chamber of the Transwell.

**Organotypic**

Fibroblast embedded gels ($2.5x10^5$ cells/ml) were prepared in DMEM with a 1:1 mixture of Matrigel (Scientific Laboratory Supplies, Nottingham, UK) and type I collagen (Merck Millipore, Billerica, Massachusetts) to create a total volume of 1ml. A mixture of $2.5x10^5$ fibroblasts and $5 \times 10^5$ cancer cells (5PT) were then plated on top of these gels. The gel was then mounted on a collagen coated nylon sheet atop a metal grid, creating an air liquid interface. Fresh KGM was applied every 2 days. After 12 days, the gels were bisected, fixed, processed to paraffin and stained with haematoxylin and eosin. Quantification of the invasive index was performed after thresholding the cellular regions in FIJI (1).

**Gel contraction Assay**

Collagen gel contraction assays were performed as described previously (5). Briefly, $2x10^5$ fibroblasts were embedded in a 1mg/ml collagen gel, which was released after setting. Contraction was monitored over a 24h period.

## RNA-Sequencing and Bioinformatics

*RNA-sequencing analysis of HFFF2s*

HFFF2 were treated with TGF-β (2ng/ml) 7 days prior to RNA extraction, RT-PCR and RNA-sequencing analysis. RNA-sequencing libraries were prepared for each RNA sample, sequenced using an Illumina HiSeq 2500 platform (Illumina, San Diego, CA, USA). The protocol employed yielded 35-bp long reads at an average sequencing depth of 42.2 million raw sequencing reads per sample (range 37.4-48.5 million). Of these reads, an average of 87.9% aligned to the hg19 reference genome file (range 85.6%-89.7%). The mappings were then converted to gene specific read count values using the script HTSeq-count (7,8), yielding read count values for a total of 23,368 annotated genes. Differential expression analysis was performed in the Bioconductor Package EdgeR importing raw counts obtained by HTSeq (7,8). From these we excluded all genes that had less than 3 read counts, giving a total of 13,094 genes. The trimmed mean of M-values (TMM) was implemented to adjust for RNA composition, followed by estimation of the biological coefficient of variation (BCV). After negative binomial model were fitted and dispersion estimates obtained, differential expression analysis was performed using generalized linear model (GLM) likelihood ratio test. This RNA-Sequencing data has been made available using the ArrayExpress archive: https://www.ebi.ac.uk/arrayexpress/ (Username: Reviewer_E-MTAB-3101; Password: qqfvtsnb)

*Comparing NOX4 expression in Normal vs Tumour samples*

RNASeqV2 Illumina Hi-seq data from patient matched normal (solid tissue) and primary tumour samples were downloaded from TCGA data matrix. Rsem normalized gene results were then log transformed and patient matched samples compared using Wilcoxon matched pairs signed rank test in Graph Pad Prism v6.

*Linear Regression analysis and myofibroblast gene signatures*

Myofibroblast/CAF gene signatures were was generated from the RNA-Sequencing analysis described above and previously described gene expression profiling experiments (9,10). These signatures were comprised of genes statistically significantly up-regulated by fibroblasts/mesenchymal stem cells treated with TGF-β/cancer cell conditioned media. The genes included in these gene signatures are detailed in **Supplementary Table 1**.

Principal component analysis was carried out to summarize the expression of myofibroblast/CAF gene signatures. RNASeqV2 Illumina Hi-seq data from primary tumour samples was downloaded from TCGA data matrix. Rsem normalized gene results were log transformed and imported into IBM SPSS Statistics v22. Principal component analysis was then carried out with the genes listed in **Supplementary Table 1** as variables, using an oblimin rotation and excluding cases in a listwise manner, the regression factor score from the 1st principal component was used as a measure of myofibroblast gene expression.

Simple linear regression analysis and assumption testing was carried out in IBM SPSS Statistics v22. In all cases the validity of this analysis was confirmed: ensuring a linear relationship between variables (examples are shown in **Supplementary Figure 3D-F**); normal distribution of residuals (mean = 0); and consistent variance was confirmed by a random distribution of residuals vs predicted values.

*Weighted Gene Correlation Network Analysis (WGCNA)*

Raw read counts from head and neck, colorectal and oesophageal cancer datasets, were imported from TCGA data portal into the R/Bioconductor package edgeR (7) and normalized using the limma-voom algorithm (11). Outlier subjects were identified and excluded using a sample network connectivity statistic described previously (12) and implemented in the R function SampleNetwork. Consensus network construction was conducted using the WGCNA package in R (13). Briefly, correlation matrices for the individual datasets were obtained by calculating the weight mid-correlations between all variable probe sets across all samples. Next, the adjacency matrix for each dataset was calculated by raising the absolute

values of the correlation matrix to a power of 12. Topological overlap (TO) matrices were then calculated, and the consensus TO between each probe pair set to the parallel minimum of the networks (14). Finally, genes were hierarchically clustered using 1-consTO as the distance measure and consensus modules identified using a dynamic tree-cutting algorithm. Networks were graphically depicted by exporting the consTO weights into the program Gephi (http://gephi.org/) and the nodes clustered using ForceAtlas **Supplementary Figure 2**. Meta-values for eigengene correlations within the consensus network were calculated by combining the Z scores of correlations from each dataset divided by the square root of the number of sets.

*NOX4 expression analysis in LCMD datasets*

NOX4 expression in NCBI GSE DATABASES was analysed using the NCBI GEO2R tool. All datasets analysed were confirmed to be median normalized. NOX4 expression data was then downloaded and sample groups were compared as described in the figure legend.

## Supplementary Figures

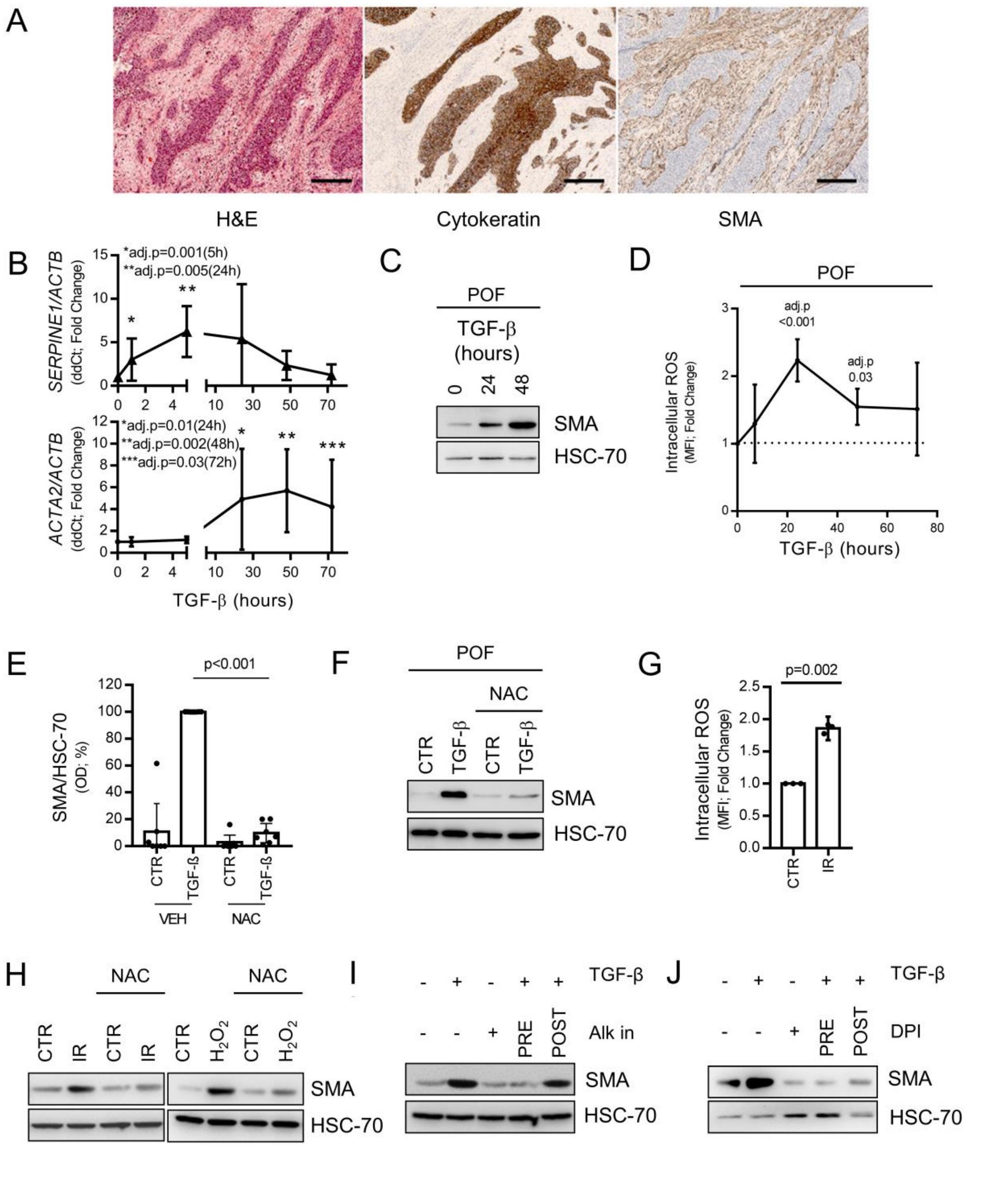


**Supplementary Figure 1: Immunohistochemistry showing myofibroblast accumulation in solid tumours, and analysis of fibroblast-to-myofibroblast transdifferentiation. A**) Representative image of High α-SMA IHC staining (HNSCC tissue), with serial sections stained for cytokeratin (IHC) and H&E. **B**) Q-PCR analysis of *ACTA2* and *SERPINE1*

expression in HFFF2s following the indicated times of TGF-β (2ng/ml) treatment (mean +/- 95%CIs from 3 independent experiments; statistical significance is shown by one-way ANOVA with Dunnet's multiple comparison test relative to the untreated control). **C-D**) Western blotting and flow cytometry analysis of DCFH-DA fluorescence respectively, from adult primary oral fibroblasts treated with TGF-β (2ng/ml) (mean +/-95%CIs from 3 independent experiments; statistical significance is shown by one-way ANOVA with Dunnet's multiple comparison test relative to the untreated control). **E**) Densitometry analysis of western blotting for α-SMA (HSC-70 loading control) from adult primary fibroblasts isolated from normal oral and colon tissues (n= 5 & 2 respectively) treated with TGF-β (2ng/ml; 72 hours) +/- NAC (5mM) (mean +/-95%CIs; statistical significance was assessed by 2-Tailed T-test with Welch's correction). **F**) Representative blot from one of the primary oral fibroblasts analysed in (**E**). **G**) Flow cytometry analysis of DCFH-DA fluorescence in HFFF2s 5 days following treatment with γ-irradiation (IR; 10Gy) or $H_2O_2$ (1mM; 1 hour pulse) (mean +/-SD, representative of a minimum of 2 independent experiments). **H**) Western blotting of HFFF2s treated with IR/$H_2O_2$ (as per (f)) +/- NAC (5mM). **I**) Western blotting of HFFF2s treated with TGF-β (2ng/ml; 72 hours) +/- an Alk5 inhibitor (1µM; TGF-βR1 inhibitor) administered either 1 hour prior to TGF-β (PRE) or 24 hours after (POST). **J**) As per (h) using diphenylene iodonium (DPI; 4µM) instead of the Alk5 inhibitor.

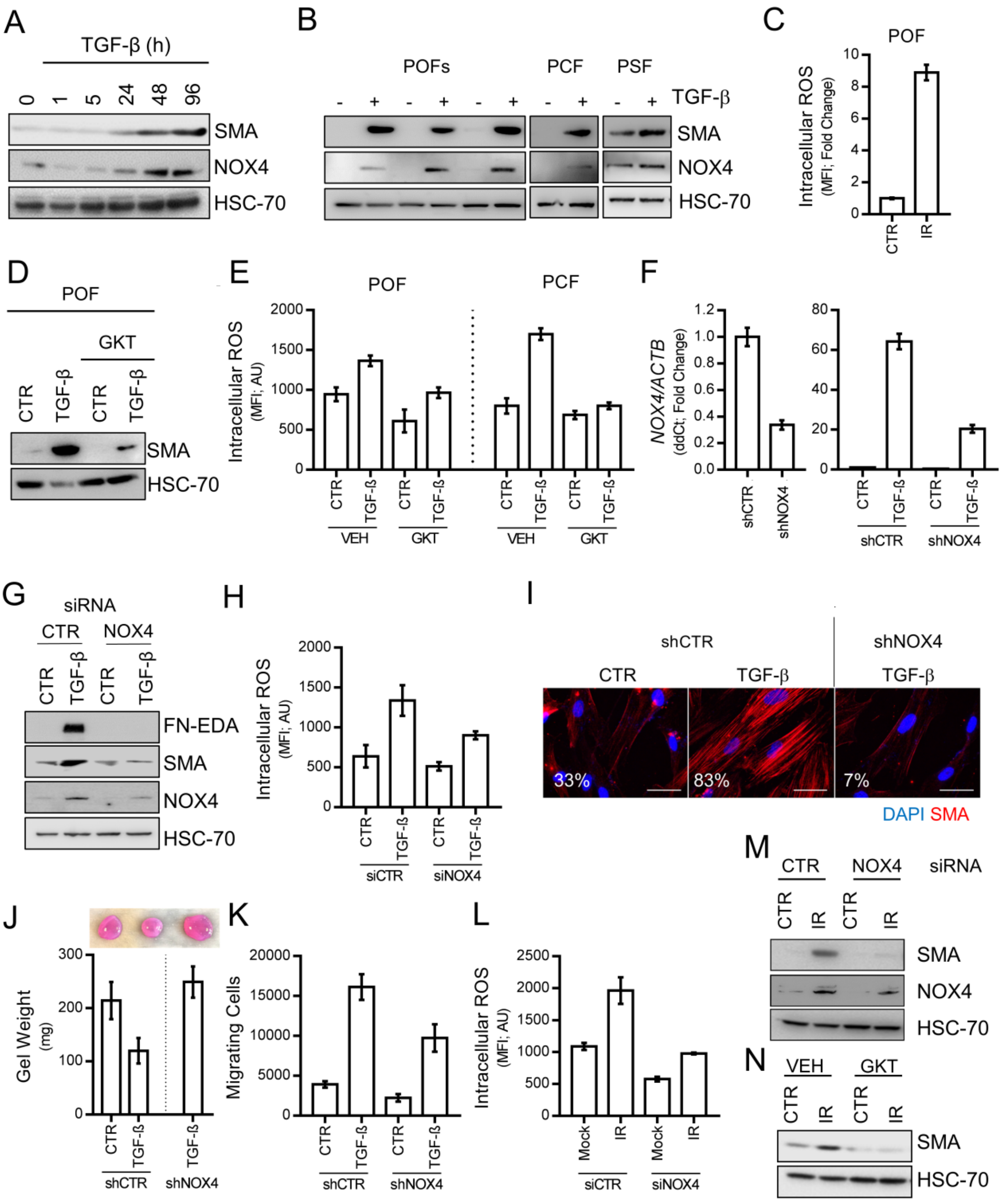


**Supplementary Figure 2: Role of NOX4 in fibroblast-to-myofibroblast transdifferentiation.** **A**) Western blotting from HFFF2s treated with TGF-β (2ng/ml) for the indicated time-periods. **B**) Western blotting analysis of adult primary oral, colon and skin fibroblasts (POF, PCF and PSFs respectively), isolated from different patients, treated with TGF-β (2ng/ml; 72 hours). **C**) Q-PCR analysis of NOX4 expression in POFs treated with

TGF-β (2ng/ml; 48 hours), (mean +/-SD from 3 technical replicates). **D**) Western blotting analysis of POFs treated with TGF-β (2ng/ml; 72 hours) +/- GKT137831 (20μM). **E**) Flow cytometry analysis of DCFH-DA fluorescence in POFs and PCFs, following treatment with TGF-β (2ng/ml; 48 hours) +/- GKT137831 (20μM) (mean +/-SD from 3 technical replicates). **F**) Representative Q-PCR analysis of *NOX4* expression in HFFF2s transduced with shRNA targeting NOX4 or a non-targeting control (CTR) (mean +/-SD from 2 technical replicates). **G**) Western blotting analysis of HFFF2s transfected with either siRNA targeting NOX4 or CTR and then treated with TGF-β (2ng/ml; 72 hours). **H**) Flow cytometry analysis of DCFH-DA fluorescence in HFFF2s transfected as per (g) and treated with TGF-β (2ng/ml; 48 hours) (mean+/-SD from 3 technical replicates). **I-K**) Immunofluorescent cytochemistry staining, gel contraction assay and migration assay respectively, with HFFF2s transduced as per (**B**) and treated with TGF-β (2ng/ml; 72 hours). **I**) the percentage of cells positive for α-SMA stress fibres is shown, Scale bar represents 50μm). **J**) Representative gel images and measurement of gel weight is shown (Mean+/-SD, 3 technical replicates). **K**) Migration of 5PT cells towards fibroblast conditioned media (mean+/-SD, 3 technical replicates). **L-M**) HFFF2s transfected with either siRNA targeting NOX4 or CTR and then treated with IR (10Gy; 72 hours). **L**) Flow cytometry analysis of DCFH-DA fluorescence (mean +/-SD of 3 technical replicates). **M**) Western blotting analysis. **N**) Western blotting analysis of HFFF2s treated with IR (10Gy; 72 hours) +/- GKT137831 (20μM).

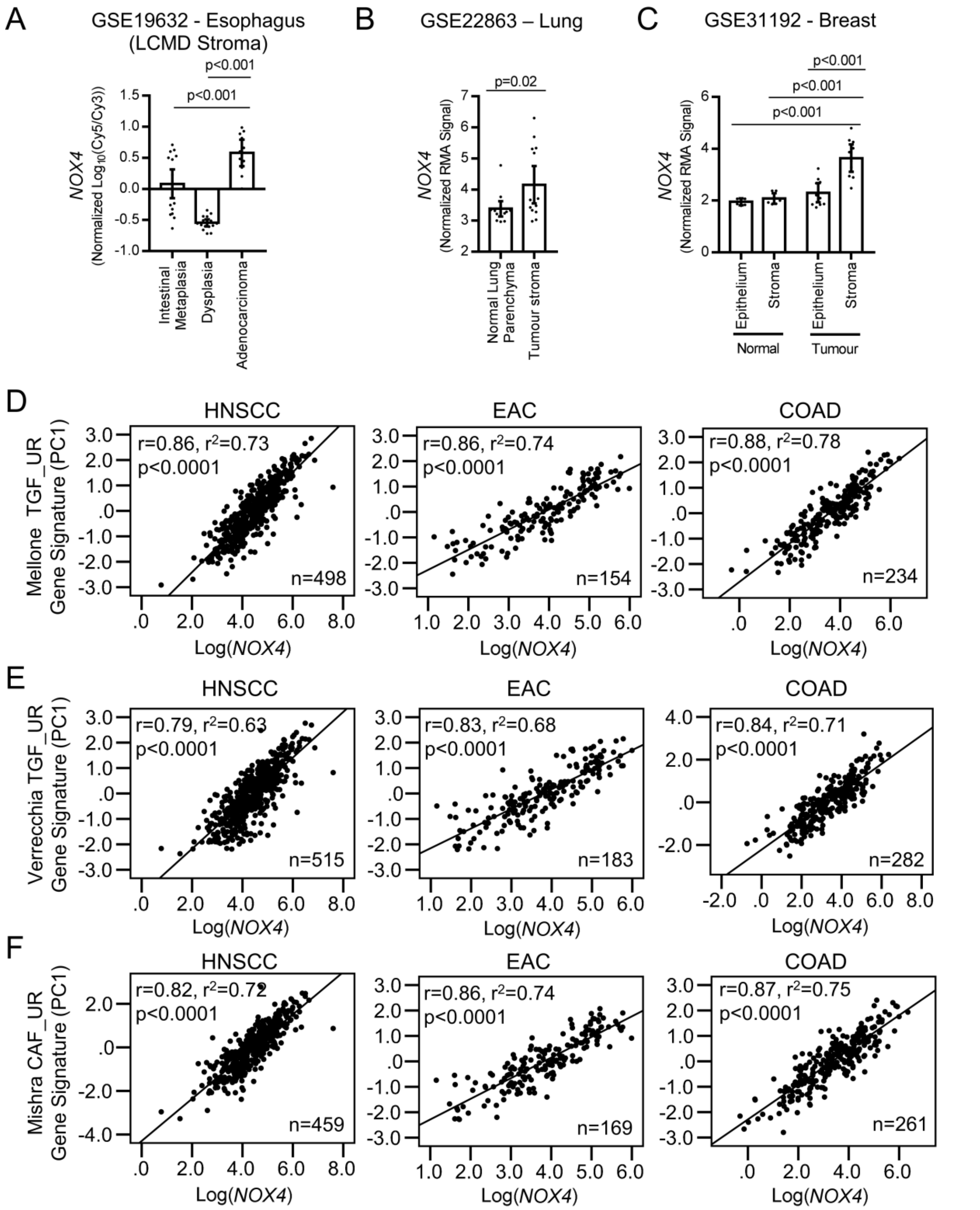


**Supplementary Figure 3: NOX4 expression in the tumour microenvironment. A-C**) Analysis of NOX4 expression in laser capture micro-dissected (LCMD) tumour and stromal compartments from the indicated tumour/tissue types. Normalised data from the NCBI-Gene expression omnibus was used to analyse NOX4 expression in the indicated GSE databases.

Data is summarised as mean +/-95%CIs and statistical significance assessed by one-way ANOVA (**A&C**) or 2-tailed T-test with Welch's correction (**B**). **D-F**) Scatter plots showing linear regression analysis of NOX4 expression and regression factor scores from the 1$^{st}$ principal component of the indicated myofibroblast/CAF gene signatures, within the HNSCC, EAC and COAD RNASeq datasets from TCGA.

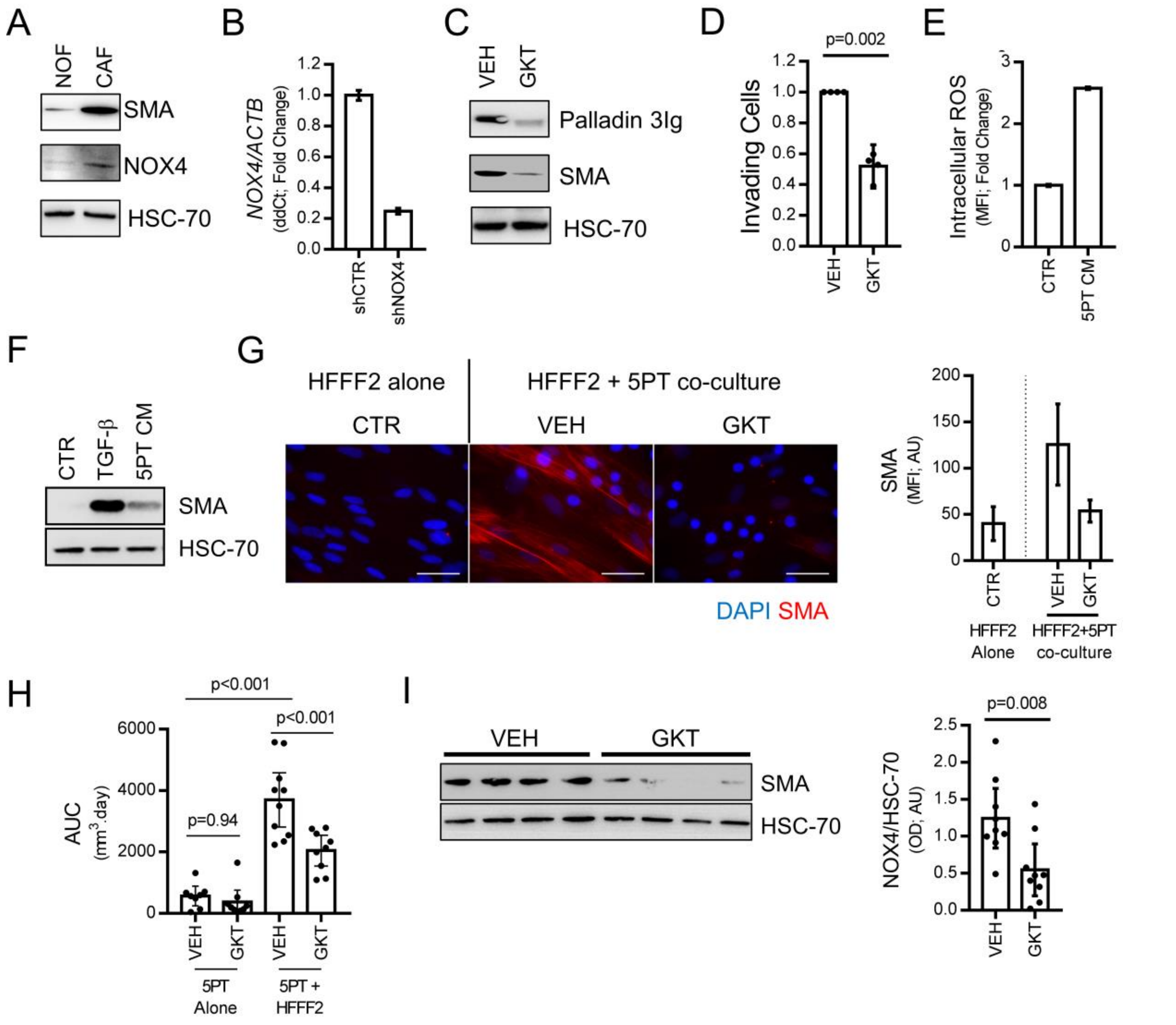


**Supplementary Figure 4: Role of NOX4 in the tumour microenvironment *in vitro* and *in vivo*.** **A**) Representative western blot for NOX4 and α-SMA in oesophageal normal fibroblasts (NOFs) and CAFs. **B**) Representative Q-PCR analysis of *NOX4* expression in HNSCC CAFs transduced with shRNA targeting NOX4 or CTR (mean +/-SD from 2 technical replicates). **C**) Western blotting analysis of EAC CAFs treated with GKT137831 (20μM; 5 days). **D**) Transwell Invasion assay where HNSCC (5PT) cells invade towards HNSCC CAF conditioned media (CM) from two independent patients (data is presented as mean +/-95%CIs from 4 independent experiments; statistical significance was assessed by 2-Tailed T-test with Welch's correction). **E**) Flow cytometry analysis of DCFH-DA fluorescence in HFFF2s treated with 5PT CM (data is presented as mean +/-SD from 3

technical replicates). **F**) Western blotting analysis of HFFF2 cells treated with 5PT CM. **G**) Immunofluorescent cytochemistry staining and quantification of α-SMA expression in HFFF2s co-cultured with 5PT cells (data is presented as the mean fluorescence intensity (MFI) +/-SD from 6 independent fields of view). **H**) 5PT xenograft tumours grown in RAG1$^{-/-}$ mice with or without HFFF2s (area under the curve (AUC) from three measurements of tumour volume per tumour (at 17, 23 & 30 days post inoculation)), each point represents a tumour (n=10 per group; mean +/-95%CIs is also shown). **I**) Representative western blot and densitometry quantification (n=10 per condition) of 5PT + HFFF2 xenograft tumours grown in RAG1$^{-/-}$ mice treated with GKT137831 (30mg/kg) by daily oral gavage, data is presented as mean +/-95% CIs. Scale bars represent 50μm.

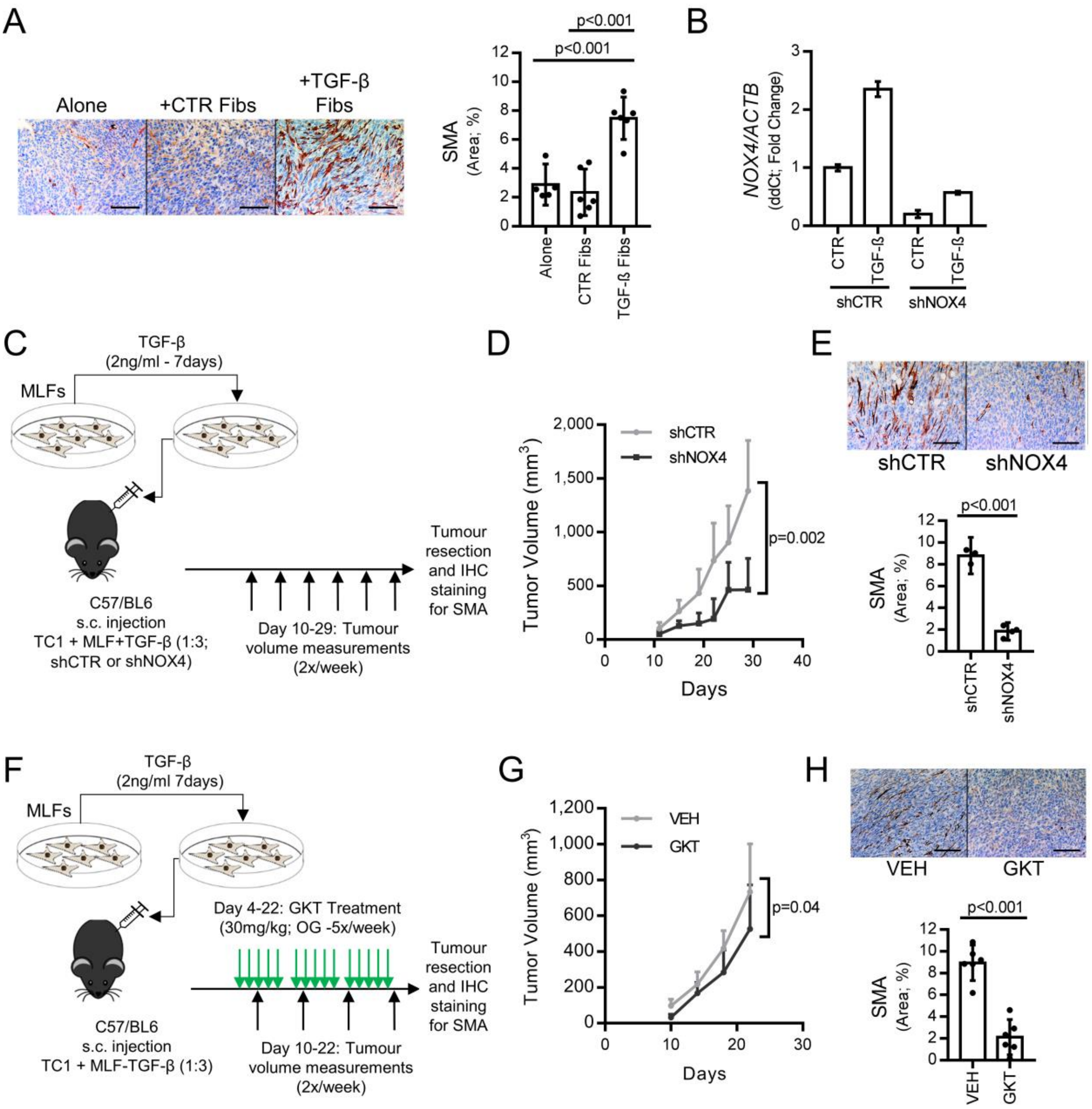


**Supplementary Figure 5: Effect of NOX4 inhibition on tumour growth and myofibroblast accumulation in an isograft murine model system.** **A**) α-SMA IHC staining of TC1 isograft tumours injected alone or co-injected with CTR MLFs or MLFs treated with TGF-β (2ng/ml; 7 days), representative images and quantification (mean +/- 95%CIs n=5-6/group; statistical significance was assessed by one-way ANOVA with Dunnett's multiple comparisons test). **B**) Representative Q-PCR analysis of NOX4 expression in MLFs transduced with shRNA targeting NOX4 or CTR (mean +/-SD from 3 technical replicates). **C-E**) TC1 + MLFs (transduced as per **Supplementary Figure 5B**)

isografts grown in C57/BL6 mice. **D**) Tumour growth curves (mean +95%CI n=6/group; statistical significance was assessed by 2-tailed homoscedastic t-test comparing the AUC). **E)** α-SMA immunohistochemistry (IHC) staining representative images and quantification (mean +/-95%CIs n=3-4/group; statistical significance was assessed by 2-tailed homoscedastic t-test). **F-H**) TC1 + MLF isograft tumours grown in C57/BL6 mice for four days and then treated with GKT137831 (30mg/kg) by daily oral gavage. **G**) Tumour growth curves (mean +/- 95%CIs n=6/group; statistical significance was assessed by 2-tailed homoscedastic T-Test comparing the area under the curve (AUC)). **H**) α-SMA IHC staining, representative images and quantification (mean +/-95%CIs n=6/group; statistical significance was assessed by 2-tailed homoscedastic T-Test). Scale bars represent 100μm.

## Supplementary Table

### Supplementary Table 1: Myofibroblast/cancer associated fibroblast (CAF) gene lists

| Gene Signature | Description | Genes |
|---|---|---|
| Mellone_TGF_UR | Top 50 most significant genes up-regulated in HFFF2s treated with TGF-β (2ng/ml) for 7 days (measured by RNA-sequencing) (15). | *ADAM12, ADM2, AMIGO2, APCDD1L, BGN, BHLHE40, CADM1, CBS, CDH2, CLSTN2, COL11A1, COL1A1, COL4A1, COL4A2, COL5A1, COL8A2, COMP, DDIT4, EFHD1, EGR2 ,ELN, FN1, HMCN1, INHBA, ITGA11, KIF26B, CD1, LRRC15, LTBP2, MFAP4, MMP11, MMP2, NIPAL4, NREP, NTM, NUAK1, ODZ3, PHGDH, POSTN, PRSS23, PTPRN, SEMA7A, SLC7A2, SLC7A5, SLIT3, SPOCK1, SULF1, TGFBI, THBS2, VCAN.* |
| Verrecchia_TGF_UR | Genes that were statistically significantly and persistently up-regulated in dermal fibroblasts treated with TGF-β (Clusters 1, 2, 4 & 6), described previously (10). | *ADAM9, ARHGDIA, BSG, CD44, CD82, CDH6, COL16A1, COL1A2, COL3A1, COL6A1, COL6A3, COL8A1, CYTH2, DSP, DVL1, EPHB2, EPHB3, EPHB4, FN1, HSPG2, ICAM1, IGFBP3, IGFBP4, ITGA3, ITGB2, JUP, LAMB1, LRP1, LRRC17, MARCKSL1, MMP1, MMP14, MMP16, MMP17, MMP3, MTA1, NEO1, NID1, NME1, NOTCH2, NOTCH2, PAK1, PAK2, PCDHGC3, PLAT, RAC1, RAC2, RHOB, RHOC, RHOG, RPSA, SERPINE1, SPARC, SSR1, TCF7L2, THBS1, THBS2, TIMP1, TIMP3, TNC, VCAN, WNT2B.* |
| Mishra_CAF_UR | Top 25 genes up-regulated by mesenchymal stem cells grown in cancer cell conditioned media, generating a CAF phenotype, described previously (9). | *BEX5, CA12, CCL2, COL6A1, COL6A2, CTHRC1, CTSK, EGR1, FGR, FOS, FXYD3, GEM, HBA2, KRT17, MMP9, PDGFRA, PDGFRB, PLAU, RAB3B, SESN2, TCN1, THY1, TMEM156, TNC.* |

## References


1. Schindelin J, Arganda-Carreras I, Frise E, et al. Fiji: an open-source platform for biological-image analysis. Nat Methods 2012;9(7):676-82.

2. Bauer JA, Trask DK, Kumar B, et al. Reversal of cisplatin resistance with a BH3 mimetic, (-)-gossypol, in head and neck cancer cells: role of wild-type p53 and Bcl-xL. Mol Cancer Ther 2005;4(7):1096-104.

3. Lewis MP, Lygoe KA, Nystrom ML, et al. Tumour-derived TGF-beta1 modulates myofibroblast differentiation and promotes HGF/SF-dependent invasion of squamous carcinoma cells. Br J Cancer 2004;90(4):822-32.

4. Naldini L, Blomer U, Gallay P, et al. In vivo gene delivery and stable transduction of nondividing cells by a lentiviral vector. Science 1996;272(5259):263-7.

5. Hanley CJ, Noble F, Ward M, et al. A subset of myofibroblastic cancer-associated fibroblasts regulate collagen fiber elongation, which is prognostic in multiple cancers. Oncotarget 2015; 10.18632/oncotarget.6740.

6. Moutasim KA, Jenei V, Sapienza K, et al. Betel-derived alkaloid up-regulates keratinocyte alphavbeta6 integrin expression and promotes oral submucous fibrosis. J Pathol 2011;223(3):366-77.

7. Robinson MD, McCarthy DJ, Smyth GK. edgeR: a Bioconductor package for differential expression analysis of digital gene expression data. Bioinformatics 2010;26(1):139-40.

8. Robinson MD, Smyth GK. Moderated statistical tests for assessing differences in tag abundance. Bioinformatics 2007;23(21):2881-7.

9. Mishra PJ, Mishra PJ, Humeniuk R, et al. Carcinoma-associated fibroblast-like differentiation of human mesenchymal stem cells. Cancer Res 2008;68(11):4331-9.

10. Verrecchia F, Chu ML, Mauviel A. Identification of novel TGF-beta /Smad gene targets in dermal fibroblasts using a combined cDNA microarray/promoter transactivation approach. J Biol Chem 2001;276(20):17058-62.

11. Ritchie ME, Phipson B, Wu D, et al. limma powers differential expression analyses for RNA-sequencing and microarray studies. Nucleic Acids Res 2015;43(7):e47.

12. Oldham MC, Langfelder P, Horvath S. Network methods for describing sample relationships in genomic datasets: application to Huntington's disease. BMC Syst Biol 2012;6:63.

13. Langfelder P, Horvath S. WGCNA: an R package for weighted correlation network analysis. BMC Bioinformatics 2008;9:559.

14. Langfelder P, Mischel PS, Horvath S. When is hub gene selection better than standard meta-analysis? PLoS One 2013;8(4):e61505.

15. Mellone M, Hanley CJ, Thirdborough S, et al. Induction of fibroblast senescence generates a non-fibrogenic myofibroblast phenotype that differentially impacts on cancer prognosis. Aging (Albany NY) 2016;9(1):114-132.
